# A pathway sensor for genome-wide screens of intracellular proteolytic cleavage

**DOI:** 10.1186/gb-2008-9-4-r64

**Published:** 2008-04-03

**Authors:** Robin Ketteler, Zairen Sun, Karl F Kovacs, Wei-Wu He, Brian Seed

**Affiliations:** 1 Center for Computational and Integrative Biology, Massachusetts General Hospital, Cambridge Street, Boston, MA 02114, USA; 2 Department of Genetics, Harvard Medical School, Cambridge Street, Boston, MA 02114, USA; 3 Origene Technologies Inc., Taft Court, Rockville, MD 20850, USA

## Abstract

Protein cleavage is a central event in many regulated biological processes. We describe a system for detecting intracellular proteolysis based on non-conventional secretion of *Gaussia *luciferase (GLUC). GLUC exits the cell without benefit of a secretory leader peptide, but can be anchored in the cell by fusion to β-actin. By including protease cleavage sites between GLUC and β-actin, proteolytic cleavage can be detected. Using this assay, we have identified regulators of autophagy, apoptosis and β-actin cleavage.

## Abstract 2

A new system based on non-conventional secretion of the luciferase from Gaussia princeps (GLUC) can be used to detect intracellular proteolysis in vivo.

## Background

Advances in automation and the availability of genomic sequence information have led to the development of sophisticated cell-based assays for high-throughput screening of functional phenotypes [1]. Most cell-based assays rely on fluorescent or luminescent reporters such as green fluorescent protein (GFP), secreted alkaline phosphatase (SEAP) or *Photinus *luciferase. Secreted luciferases offer many advantages over cellular reporter enzymes as they can be non-destructively harvested from cellular supernatants over time. Several secreted luciferases have been reported, from the marine copepods *Gaussia princeps *[2], and *Metridia longa *[3], the ostracod *Vargula hilgendorfii *[4], the decapod shrimp *Oplophorus gracilirostris *[5] and the ostracod crustacean *Cypridina noctiluca *[6]. In addition, intracellular luciferases, such as from the sea pansy *Renilla reniformis*, can be engineered to be secreted and stable in the extra-cellular milieu [7].

A cDNA encoding *G. princeps *luciferase (GLUC) activity has recently been isolated and found to direct the synthesis of a 19.9 kDa protein that has utility as a bioluminescent reporter [2]. GLUC can be used to monitor *in vivo *processes and can be easily harvested from biological fluids such as blood or urine [8]. Assays based on GLUC activity have been used to study, among other topics, processing through the secretory pathway [9], the strength of signal peptides [10], endoplasmic reticulum (ER) stress [11], DNA hybridization [12], and protein-protein interaction using complementary fragments derived from the enzyme [13]. By deletion of the signal peptide, a GLUC mutant has been engineered for monitoring *in vivo *gene expression; very low bioluminescence was detected in cell culture superanatants upon expression of this construct [2]. However, overall bioluminescence of this construct was greatly reduced compared to wild-type GLUC [2]. It has been noted that GLUC is secreted when fused to the ER retention signal KDEL, which has been attributed to changes in the protein conformation or processing in the ER and Golgi [2].

We have generated a GLUC variant that is secreted in the absence of a signal peptide. We present here a cell-based assay for the detection of general protease activity based on inducible luciferase secretion. GLUC can be anchored in cells by fusion to β-actin. Insertion of protease cleavage sites in a linker between β-actin and GLUC allows monitoring the cleavage of short peptides, as well as cleavage of native full-length proteins of any sequence inserted. We present GLUC-based reporter systems for monitoring apoptosis and autophagy and describe applications of this reporter in genome-wide screening approaches.

## Results

In the course of attempts to develop a GLUC reporter that is retained in cells and released after addition of a specific stimulus, we deleted the signal peptide to generate dNGLUC. Surprisingly, this deletion did not abolish the accumulation of GLUC activity in the supernatant (SN) of transiently transfected 293ET cells. Although the proportion of dNGLUC in SN was reduced to 30.5% compared to 96.7% of total GLUC activity, the overall activity was still very high (Table 1). By contrast, when dNGLUC was fused to the carboxyl terminus of β-actin, less than 1.5% of GLUC activity was detected in SN (Table 1), and the relative light unit values observed were close to background (not shown).

**Table 1:** dNGLUC is secreted in the absence of a signal peptide

	% secreted
GLUC	96.7 ± 27.6
dNGLUC	30.5 ± 7.8
Actin-dNGLUC	1.5 ± 0.4

GLUC activity was determined in SN and whole cell lysate of 293ET cells transfected with the indicated constructs. The percentage of secreted *Gaussia *luciferase activity was calculated from three independent transfections.

**Figure 1:**
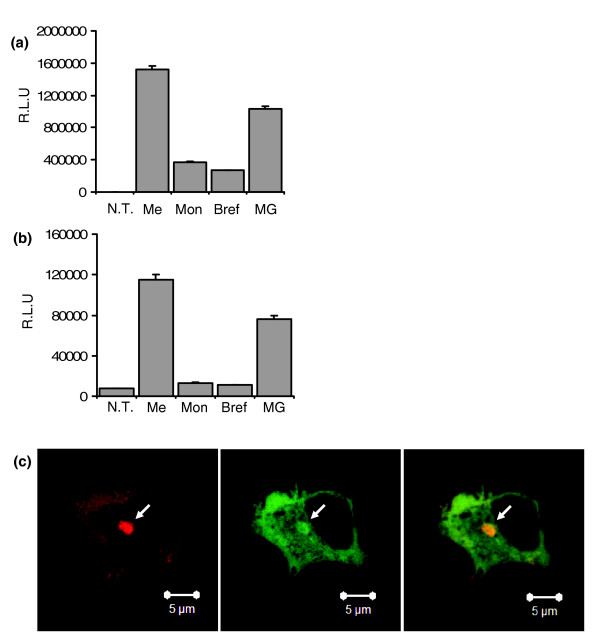
dNGLUC secretion is Monensin and Brefeldin A-sensitive. **(a, b) **Monensin and Brefeldin A inhibit non-conventional secretion of dNGLUC (a) and secreted alkaline phosphatase (SEAP) (b). Twenty-four hours after transfection of 293ET with dNGLUC, medium was replaced with medium containing 7 μM Monensin (Mon), 10 μg/ml Brefeldin A (Bref) and 5 μg/ml MG132 or Methanol (Me). SN was collected after 4 h prior to analysis of GLUC or SEAP activity in the supernatant. NT, not transfected; RLU, relative light units.) **(c) **Co-localization of GFP-dNGLUC and Golgin-67. 293ET cells were transfected with GFP-dNGLUC and Golgin67-DsRed and cells were fixed with 4% paraformaldehyde prior to analysis by confocal microscopy. Overlap of GFP-dNGLUC and Golgin67-DsRed is marked by an arrow.

Most extracellular proteins are secreted from cells by transport through a secretory pathway that requires translocation of the nascent polypeptide from the ribosome to the lumen of the ER, followed by vesicular transport through the Golgi and subsequent compartments [14]. Initiation of secretion by this pathway is mediated by a hydrophobic amino-terminal signal sequence [14]. Some proteins, however, lack an amino-terminal signal peptide and are secreted by a mechanism that is insensitive to treatment with inhibitors of ER/Golgi trafficking such as Brefeldin A [15,16]. To further characterize the mechanism of secretion of dNGLUC, we treated 293ET cells expressing dNGLUC with drugs known to interfere with secretory pathways. Cells expressing dNGLUC were exposed to 7 μM Monensin, 10 μg/ml Brefeldin A or 5 μg/ml MG132 and the activity accumulating over 4 h at 37°C was determined (Figure 1a). For comparison, we also measured the activity of SEAP, which is secreted by a classical signal peptide (Figure 1b). We found that treatment with Monensin and Brefeldin A reduced secretion of both dNGLUC (by 75% and 82%, respectively; Figure 1a) and SEAP (by 88% and 90%, respectively; Figure 1b), while MG132, an inhibitor of the proteasome, reduced secretion by 32%. Since Monensin and Brefeldin A interfere with transport pathways originating from the Golgi apparatus, we propose that dNGLUC is secreted by a mechanism involving the secretory pathway. To confirm this hypothesis, we performed co-localization studies of dNGLUC and the Golgi marker protein Golgin-67. GFP-tagged dNGLUC was localized in the cytoplasm. In addition, we observed co-localization of GFP-dNGLUC and DsRed-tagged Golgin-67 at a perinuclear site (Figure 1c). Co-localization with Golgi-resident proteins is consistent with the view that secretion of dNGLUC requires ER/Golgi trafficking. As far as we are aware, the dNGLUC secretory pathway is presently the first example of Brefeldin A-sensitive non-conventional secretion.

**Figure 2:**
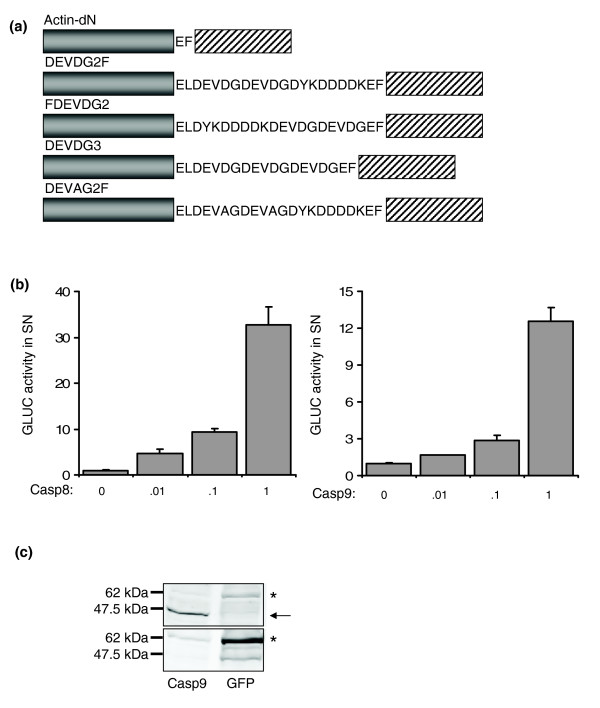
Design of a GLUC-based caspase sensor. **(a) **Schematic representation of Actin-dN, DEVDG2F, FDEVDG2, DEVDG3 and DEVAG2F. Actin, grey box; dNGLUC, shaded box. **(b) **Activation of FDEVDG2 by caspase 8 and 9. 293ET cells were co-transfected with 500 ng of Actin-dN or FDEVDG2 and the indicated amounts of caspase 8 (left panel) or caspase 9 (right panel) in a 12-well plate. SN was tested for GLUC activity after 30 h. Error bars were calculated from three independent transfections. RLU, relative light units. **(c) **Immune-blotting of cleaved FDEVDG2. Transiently transfected 293ET cells expressing FDEVDG2 together with GFP or caspase 9 were grown for 30 h prior to cell lysis. Lysates were resolved by 10% PAGE and immune-blots were analyzed with anti-Flag M2 (upper panel) or anti-GLUC (lower panel) antibody. Full-length FDEVDG2 migrates at 62 kDa (marked by an asterisk) and caspase 9-cleaved Actin-FDEVDG2 migrates around 46 kDa (marked by an arrow).

The intracellular retention of Actin-dNGLUC opened the possibility of developing an assay for release of dNGLUC by protease-mediated cleavage. We inserted two tandem repeats of the caspase 9 protease consensus site DEVDG and a Flag tag between β-actin and dNGLUC to generate two candidate caspase sensors, Actin-DEVDG2-flagdNGLUC (DEVDG2F) and Actin-flagDEVDG2-dNGLUC (FDEVDG2) (Figure 2a). In co-transfection experiments in which variable amounts of caspase 8 or caspase 9 expression plasmid were co-delivered with a fixed amount of dNGLUC expression plasmid, dNGLUC activity was released into SN in a dose-dependent manner with increasing amounts of expression plasmid (Figure 2b). Luciferase activity in the SN was 4.8-fold, 9.3-fold and 32.8-fold higher in the presence of 10 ng, 100 ng and 1,000 ng of caspase 8, respectively, compared to control cells transfected with vector alone. Expression of caspase 9 resulted in 2.8-fold and 12.6-fold increases of released luciferase activity from FDEVDG in the presence of 100 ng and 1,000 ng of caspase 9, respectively (Figure 2b). We did not observe release of dNGLUC from cells expressing Actin-dNGLUC in the presence of caspase 8 or 9, confirming the specificity of cleavage for the DEVDG motif. In order to visualize the cleavage products, cell lysates from 293ET cells expressing FDEVDG2 were resolved by SDS-PAGE and immuno-blotted with anti-Flag antibodies. A band was detected at a size of 62 kDa, which corresponds to the calculated molecular size (61.5 kDa) of FDEVDG2 (Figure 2c, upper panel). In the presence of caspase 9, full-length FDEVDG2 was not detected, but a band at 46 kDa appeared, consistent with the removal of dNGLUC. Probing of the same blot with an antibody raised against GLUC identified a major band at 62 kDa that disappeared upon expression of caspase 9 (Figure 2c, lower panel).

**Figure 3:**
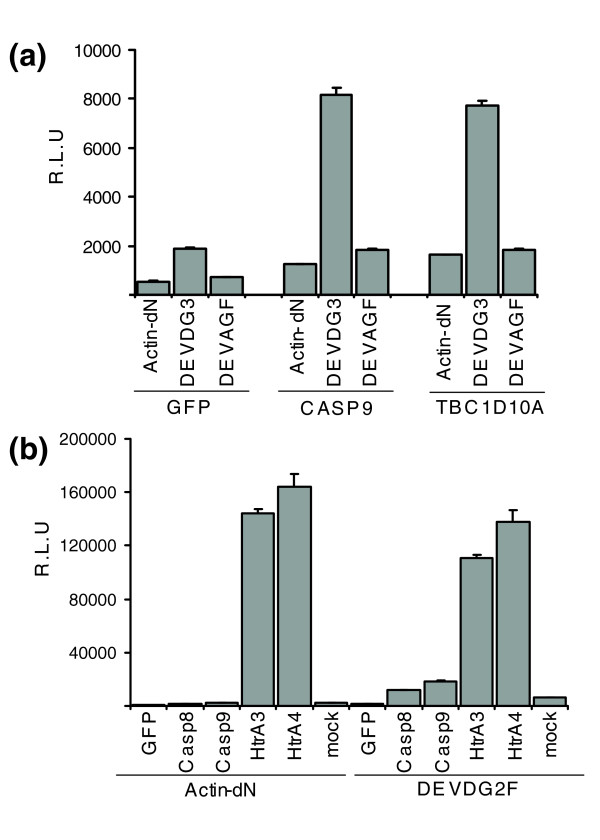
HTRA3 and 4 release GLUC activity from Actin-dNGLUC. **(a) **Actin-dNGLUC, DEVDG3 or DEVAG2F were co-transfected with GFP, caspase 9 or TBC1D10A and GLUC activity in SN was assayed after 30 h. TBC1D10A specifically releases dNGLUC from DEVDG3, but not DEVAG2F (RLU, relative light units). **(b) **HTRA3 and 4 release GLUC activity from Actin-dNGLUC. Caspase 8, caspase 9, HTRA3 and HTRA4 were co-transfected with Actin-dNGLUC and DEVDG2F and SN were analyzed for GLUC activity in SN after 30 h. HTRA3 and 4 release GLUC activity from Actin-dN and DEVDG2F, while caspases 8 and 9 released GLUC activity from DEVDG2F but not Actin.

In order to identify novel proteins that might induce caspase-mediated cleavage, we performed a functional screen using the Origene Trueclone™ expression vector collection. We co-transfected 96-well plates with single cDNA expression vectors and DEVDG2F and measured luciferase activity in SN and cellular lysates in triplicate plates. To normalize for cellular expression and cell numbers, we determined the ratio of luciferase activity in SN over cellular lysates from the same 96-well plate. Three wells on each plate were transfected with DEVDG2F only to determine the level of background secretion. In Table 2, we summarize genes that showed more than a three-fold increase in GLUC activity released from cells expressing DEVDG2F compared to cells transfected with reporter only. The candidates found include known inducers of apoptosis, such as BAK, FADD, BAD and caspase 8, in partial validation of the approach to identify regulators of caspase activation. In addition, we identified the novel genes for ASPH, PIR121, PERP and TBC1D10A, which induced 14.2-, 12.1-, 10.4- and 5.5-fold increases in GLUC activity in SN from DEVDG2F cells, respectively (Table 2). TBC1D10A is a member of the Tre/Bub2/Cdc16 (TBC) family that exhibits GTPase activating protein (GAP) activity and, thus, is an interesting candidate gene in the context of apoptotic signaling. Since DEVDG2F harbors additional aspartate residues within the Flag peptide sequence that might serve as cleavage target sites, we also generated a construct with three DEVDG-repeats without a Flag tag, Actin-DEVDG3-dNGLUC (DEVDG3). In addition, we generated a variant reporter in which the DEVDG-motif was replaced with a DEVAG motif that is not a substrate for caspases. TBC1D10A was co-transfected with Actin-dNGLUC, DEVDG3 or DEVAG2F and the release of GLUC into SN was measured. Caspase 9 induced a 4.1-fold and TBC1D10A a 4.3-fold increase in extra-cellular GLUC activity compared to GFP, but did not release dNGLUC from Actin-dNGLUC or DEVAG2F (Figure 3a). These results are consistent with the view that the cleavage promoted by caspase 9 and TBC1D10A is specific to the caspase cleavage site introduced in the reporter substrate.

**Table 2:** Genes that induce release of dNGLUC activity in SN

Gene	Fold ratio SN/cells
*ASPH*	14.2 ± 5.8
*BAK1*	12.5 ± 2.3
*PIR121*	12.1 ± 2.7
*PERP*	10.4 ± 2.5
*TBC1D10A*	5.5 ± 2.7
*FADD*	4.9 ± 1.8
*BAD*	4.8 ± 2.7
*CASP8*	3.2 ± 1.1

Cells were transfected in 96-well plates with DEVDG2F and cDNA expression vectors from the Origene Trueclone™ collection in triplicates. Activity of GLUC was measured in SN and cell lysates after 26 h and ratios of SN/cellular activity were calculated for each plate. Three wells on each plate were transfected with reporter only to determine the background activity. We show the fold ratio of SN/cellular activity over background averaged from three plates.

In the course of the screen we also identified genes that induce release of dNGLUC from Actin-dNGLUC. Co-expression of the serine peptidase HTRA4 with ActindNGLUC or DEVDG2F yielded a 201.5-fold increase of GLUC activity in SN from cells expressing Actin-dNGLUC and a 110.8-fold increase from DEVDG2F, indicating that the caspase cleavage site is not required for liberation of luciferase activity (Figure 3b). Similarly, another family member, HTRA3, induced a 177.1-fold and 89.1-fold increase in GLUC activity in SN for Actin-dNGLUC and DEVDG2F, respectively. Caspases 8 and 9 induced a 9.5-fold and 15.0-fold increase of GLUC activity for DEVDG2F, but had no effect on Actin-dNGLUC. In accordance with previous reports that have identified HTRA2-mediated cleavage of β-actin by mass spectroscopy [17], these data support the view that HTRA3 and 4 cleave within the β-actin sequence. We therefore conclude that our assay also allows the detection of full-length protein cleavage under physiological conditions.

**Figure 4:**
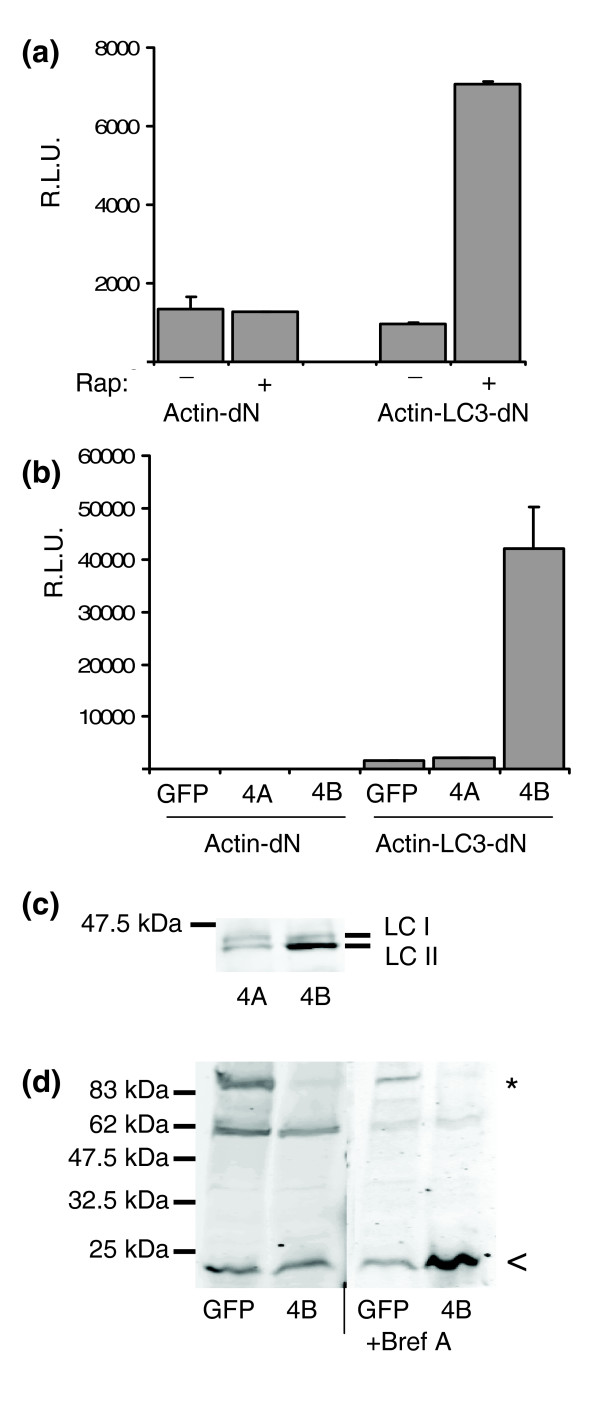
A GLUC-based sensor to monitor autophagy. **(a) **Rapamycin (Rap) induces release of GLUC activity from Actin-LC3-dN. Actin-LC3-dN was transfected in 293ET cells and medium was replaced after 24 h with serum-free medium containing 200 nM rapamycin for 6 h before analysis of GLUC activity in SN. **(b) **ATG4B but not ATG4A induces cleavage of Actin-LC3-dN. SN of 293ET cells transiently co-transfected with Actin-dN or Actin-LC3-dN and GFP, ATG4A or ATG4B were collected after 24 h and analyzed for GLUC activity. Error bars were calculated from three independent transfections. RLU, relative light units. **(c) **ATG4B cleaves GFP-LC3. 293ET cells transfected with GFP-LC3 and ATG4A or ATG4B were lysed in 1% NP40, resolved by 10% SDS-PAGE and blotted with anti-GFP. Full-length GFP-LC3 (LC I) runs at 45 kDa and the cleaved product runs at 43 kDa (LC II). **(d) **ATG4B cleaves Actin-LC3-dN to generate a small LC3-dNGLUC fragment. 293ET cells transfected with Actin-LC3-dN and GFP or ATG4B were treated for 6 h with 10 μg/ml Brefeldin A (right panel) to block secretion of cleaved dNGLUC or left untreated (left panel) before lysis in 1% NP40. Whole cell lysates were resolved by 10% SDS PAGE and blotted with an antibody raised against dNGLUC. The protein band corresponding to full-length Actin-LC3-dN is marked with an asterisk, the cleavage product is marked with an arrowhead.

To further explore the suitability of GLUC fusions for detection of native protein cleavage, we inserted the open reading frame of hMAP1LC3 (LC3), a marker of autophagy, between β-actin and dNGLUC. Autophagy is a tightly regulated cellular response to starvation that results in degradation of subcellular organelles. LC3 is cleaved during autophagy at the carboxyl terminus by the cellular protease hATG4B; the cleaved form is found associated with autophagosomes [18]. Amino acid starvation or treatment with rapamycin is sufficient to induce autophagy and LC3 cleavage. Upon treatment with 200 nM rapamycin, we detected a 7.2-fold increase in GLUC activity in SN in cells expressing Actin-LC3-dNGLUC but not Actin-dNGLUC (Figure 4a). In addition, co-expression of the cellular protease ATG4B, but not ATG4A or GFP, resulted in a 26.6-fold increase in extra-cellular luciferase activity (Figure 4b). Activity of ATG4B was confirmed by immunoblotting of transfected GFP-LC3. In the presence of ATG4B, the cleaved form of GFP-LC3 was visualized at 43 kDa and significantly increased in intensity compared to the full-length 45 kDa form, which was not evident in cells transfected with ATG4A (Figure 4c). In order to confirm cleavage of Actin-LC3-dNGLUC by ATG4B, we resolved whole cell lysates by SDS-PAGE and immuno-blotting using an antibody raised against dNGLUC as a probe. In the absence of ATG4B, we detected the full-length construct Actin-LC3-dNGLUC at 83 kDa and a smaller band at 23 kDa (Figure 4d). In cells cotransfected with an ATG4B expression plasmid, the full-length product at 83 kDa disappeared; whereas in cells cotransfected with a GFP expression plasmid, the 83 kDa product was readily apparent (Figure 4d). To visualize the secreted cleavage product, we treated cells with Brefeldin A for 6 hours prior to cell lysis. In the setting of ATG4B coexpression in Brefeldin A-treated cells, the band corresponding to Actin-LC3-dNGLUC at 83 kDa is not seen and the 23 kDa band corresponding to the dNGLUC cleavage product has increased intensity, consistent with the view that Actin-LC3-dNGLUC is cleaved by ATG4B.

**Figure 5:**
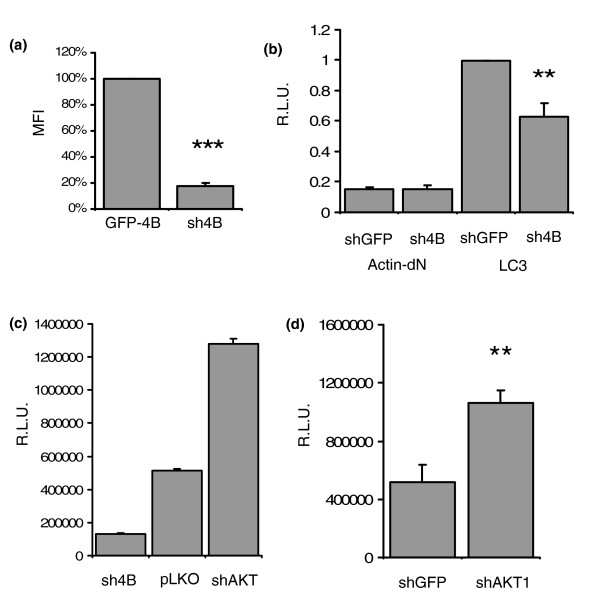
shRNA targeting AKT1 enhances autophagy. **(a) **shRNA targeting human ATG4B (sh4B) reduces expression of a GFP-ATG4B fusion protein. 293ET cells expressing GFP-ATG4B (GFP-4B) with vector control or shRNA targeting ATG4B were analyzed for mean fluorescence intensity (MFI) by FACS 48 h after transfection. MFI is given as percentage of the control cell population (vector only). **(b) **sh4B reduces basal levels of dNGLUC release from cells expressing Actin-LC3-dN. 293ET cells were transfected with Actin-LC3-dN and sh4B or a control shRNA targeting GFP and released dNGLUC activity in the SN was detected after 48 h. Control cells express Actin-dN and shGFP. Error bars were calculated from three independent transfections **(c) **shRNA mediated knockdown of AKT1 induces dNGLUC release from Actin-LC3-dN. 293ET cells expressing Actin- LC3-dN with shATG4B, shAKT1 and vector control were cultured for 48 h prior to collection of SN and analysis of dNGLUC activity. **(d) **Generation of stable 293ET cells. 293ET cells were transduced with Actin-LC3-dN and selected at 0.3 μg/ml puromycin. Seventy-two hours after transfection with shRNA targeting GFP or AKT1, dNGLUC activity was determined in the supernatant from four independent trasnfections. RLU, relative light units. (Significances were calculated by a two-sided paired ttest as marked by asterisks: **, p < 0.01; ***, p < 0.001)

To study the role of endogenous proteins in the autophagy pathway, we used small hairpin RNA (shRNA)-mediated knockdown of candidate signaling molecules. We first identified a shRNA sequence targeting human ATG4B (sh4B) that significantly reduced the expression of a GFP-ATG4B construct by 80% of detected GFP mean fluorescence intensity in 293ET cells (Figure 5a). The dNGLUC activity in the SN of 293ET cells cotransfected with Actin-LC3-dNGLUC and sh4B was reduced by 40% compared to cells transfected with Actin-LC3-dNGLUC alone, indicating that endogenous ATG4B contributes to the observed luciferase release (Figure 5b). Next, we tested the effect of shRNA-mediated knockdown of AKT1, an upstream kinase that activates the key inhibitor of autophagy, mTOR [19]. Knockdown of AKT1 resulted in an increase of dNGLUC release from cells expressing Actin-LC3-dNGLUC compared to vector control in transient transfection as well as in a stable 293ET cell line expressing Actin-LC3-dNGLUC transfected with shAKT1 (Figure 5c,d). The magnitude of the effect of shRNA knockdown was similar to that observed following inhibition of mTOR with rapamycin (Figure 4a).

## Discussion

### Non-conventional secretion of *Gaussia *luciferase

Protein secretion in most cells is mediated by signal sequences that target the nascent polypeptide chain of the elongating translation product to a secretory pore in the ER [14]. Within the ER and the subsequent compartments of the Golgi apparatus, folding and post-translational modifications take place, and the mature, modified polypeptide is released into the extracellular space. A number of secreted proteins that do not utilize the ER membrane translocation machinery, such as fibroblast growth factor, coagulation factor XIII and interleukin-1β are secreted by a non-conventional secretory pathway [16]. Different mechanisms for non-conventional secretion have been proposed [16], including lysosomal secretion for interleukin-1β [20], a plasma resident transporter for fibroblast growth factor 2 [21] and cell injury for coagulation factor XIII [22]. Two prevalent features of non-conventional secretion are the absence of a signal peptide and insensitivity to Brefeldin A [15]. The precise mechanism of secretion is still poorly understood and the underlying molecular signals remain to be elucidated.

The luciferase release assay reported here relies on a non-conventional secretion of dNGLUC that is inhibited by Monensin and Brefeldin A. Monensin inhibits acidification of terminal compartments thought to lie immediately prior to extracellular release, whereas Brefeldin A inhibits ER-to-Golgi transport. The amino-terminal amino acid sequence of the deleted luciferase studied here does not fulfill the accepted criteria for a signal peptide [23]. Because secretion is sensitive to treatment with Brefeldin A, we conclude that a previously unarticulated mechanism is responsible for the translocation of the polypeptide into the ER and/or Golgi. The molecular basis of this translocation, and subsequent passage through the terminal secretory apparatus, is presently under investigation. A Golgi-resident protein, GRASP, has been identified that is required for a non-conventional secretory pathway in *Dictyostelium discoideum *[24] and *Drosophila melanogaster *[25], and that is a candidate for mediating Brefeldin A-sensitive secretion of dNGLUC. Identification of GLUC mutants that are retained inside cells may help to identify the mechanism of non-conventional secretion.

### A novel protease assay

The present assay system has several advantages over existing systems for measuring protease activity. Currently, protease cleavage sites can be inferred from comparison of primary sequences. The physiological relevance of predicted cleavage sites in a particular protein then can be assessed by further experimentation. Target motifs can be identified by analysis of protease action on peptide libraries, such as phage display libraries, positional-scanning libraries and mixture-based libraries [26]. The identification of protein cleavage in the context of live cells can be achieved by mass spectroscopic analysis of cleavage products [27], but requires a complex experimental setup and is not amenable to high-throughput approaches. Other cell-based protease assays rely on generation of a fluorogenic substrate upon cleavage, but these assays are not genetically encoded, thus limiting their applicability *in vivo*. Some *in vivo *protease assays have been developed that exploit the properties of fluorescence resonance energy transfer (FRET) [28]; in these, the protease-mediated separation of a donor and acceptor fluorophore results in changes of the ratio of fluorescence intensities at different wavelengths [29]. A major advantage of FRET-based methods is their ability to provide information about the sub-cellular localization of protease activity. However, FRET-based assays are frequently not highly sensitive, require a carefully characterized cohort of control samples in a single experiment and typically demand advanced instrumentation. To date there has been little use of FRET in genomic screening applications. In contrast, the assay system described here non-invasively measures protein cleavage over time in the context of the complex physiology of intact living cells, is compatible with high-throughput screening methodologies, and can be designed to monitor protease function with high specificity. The luciferase release system can detect cleavage of short peptides as well as cleavage of full-length proteins. Evaluation of actin-specific or non-specific screening hits can be identified and eliminated by secondary screening with a luciferase fusion bearing a mutated version of the protease cleavage motif to be investigated. It has previously been established that GLUC secretion is proportional to cell number [2]. Differences in cell number as well as variation in transcription and translation rate can be assessed by determining ratios of extracellular luciferase to cellular activity. We recommend that the optimal harvest and collection times be assessed in pilot studies. For instance, extensive cell death results in reduced reporter production, and in the case of the apoptosis sensor used here, best results were seen when the cultures were assayed 24-32 hours after initiation of apoptosis. The assay is highly reproducible following transient transfection, and can also be used in cell lines stably transfected with the reporter if desired. Both transfected and endogenous protease activities are easily detected with this system. The transfer of a reporter enzyme across cell membranes constitutes an unexpected assay principle that adds a flexible, broadly applicable approach to current cell-based multi-color and multienzyme assays.

### Applications of the protease sensor to study β-actin cleavage, apoptosis and autophagy

Cleavage of Actin-dNGLUC by HtrA3 and 4 suggests that members of the HtrA family of heat shock proteases, which are known to have significant functions in protein folding and apoptosis, may have the general property of cleaving actin in a manner that eliminates its ability to form insoluble fibers. Recently, a proteomic approach based on mass spectroscopic identification of cleavage products was undertaken to identify HTRA2 substrates [17]. Major cleavage products included β-actin and tubulin alpha/beta and it was suggested that HTRA2 regulates apoptosis at the level of the cytoskeleton [17]. Although β-actin has been reported as a substrate for a number of caspases, including caspase 3 [30], we have not observed release of dNGLUC from Actin-dNGLUC in response to caspase 3, 8 or 9, suggesting either that cleavage did not occur, or that it did not impair the ability of β-actin to anchor dNGLUC in the cell. In contrast to observations on cell-free extracts, cleavage of β-actin by caspases has not been detected in intact cells [31].

In a functional screen using the caspase sensor, we have identified the TBC family member TBC1D10A as an inducer of DEVDG-mediated cleavage. The TBC family of proteins exhibit GAP activity towards small GTPases of the Rab family [32]. TBC1D10A has recently been identified as a GAP for Rab27A, suggesting a role in melanocyte transport and secretion [33]. In addition, TBC1D10A binds to a complex of EBP50 with Ezrin and ARF6-GTP to regulate microvillus structure [34]. Based on these data, TBC1D10A has been proposed as a regulator of protein trafficking in cells. Recently, a genome-wide screen for cell death effectors identified another family member, TBC1D10C, as an inducer of apoptosis [35]. In agreement with this observation, our findings confirm a role for TBC1D10A as an effector of protein cleavage.

Autophagy is an essential cellular process for the degradation of proteins and organelles that has been associated with neurogenerative diseases, cancer and infection [36]. Although autophagy is currently widely investigated, the systematic identification of molecular events in autophagy has been hampered by the lack of suitable assays. Current assays to study autophagy measure the accumulation of autophagic vacuoles by staining with fluorescent dyes such as monodansylcadaverine [37], or the sequestration of radioactive sugars or enzymes such as lactate dehydrogenase [38]. However, these assays are difficult to quantify due to the presence of background levels of autophagic vacuoles or non-specific staining. Recently, immuno-blotting of hMAP1LC3 cleavage products, and GFPhMAP1LC3 translocation to autophagosomes [18] have been proposed as specific assays for autophagy. However, since the cleavage product of hMAP1LC3 is itself degraded by autophagy, interpretation of these assays requires additional controls [39]. The assay presented here is a simple, easily implemented, quantitative assay that measures induction of autophagy without destruction of the cell being studied. As such, we anticipate it will be useful to many investigators in their studies of this enigmatic process.

## Conclusion

It has been estimated that the human genome contains more than 500 proteases [40], most of which are poorly characterized. The luciferase secretion assay described here can be used to identify protease regulatory pathways as well as protease targets. The actions of nongenomic proteases, such as the HIV or HCV proteases or Anthrax lethal factor can be easily assessed by inserting the appropriate peptide target sequence in an actin-peptide-dNGLUC reporter construct.

The finding that *Gaussia *luciferase is capable of exiting the cell by a non-conventional secretion pathway is unusual in itself, and provides a tool to explore aspects of non-conventional secretion. Regulated non-conventional secretion of an enzymatic reporter has not been previously demonstrated to our knowledge, and affords several advantages over existing methods for analysis of intracellular cleavage events.

Of particular interest is the process of autophagy. Autophagy is a highly regulated process that appears to provide additional energy to cells under conditions of starvation. Autophagy has been suggested to play roles in the prevention and progression of cancers [41]. The precise role that autophagy plays in these settings is not well understood, and high interest is currently directed toward understanding the contribution of autophagy to tumor growth. Large-scale screening approaches to identify regulators of autophagy to date have not been reported, possibly due to the absence of suitable screening assays. Analysis of autophagy is presently based on qualitative ultramorphological analyses, immunoblotting, or translocation of GFPLC3. Such assays can be non-quantitative, laborious and subject to multiple confounding factors [39]. The analysis system described here facilitates insight into the regulation of autophagy and enables large scale shRNA knockdown and expression screening approaches.

## Materials and methods

### Plasmids

A GLUC sequence optimized for expression in both *Escherichia coli *and *Homo sapiens *was synthesized by tandem DNA oligonucleotide annealing and sub-cloned into pEAK12. During this process, the carboxy-terminal amino acid sequence LYK was added. Human β-actin was amplified from Origene Trueclone™ (AB1024H03) and inserted into the *Hin*dIII and *Not*I sites in pEAK12 using primers 5'-GACAAGCTTATGGATGATGATATCGCC-3' and 5'-GACGCGGCCGCTTAGAATTCGAAGCATTTGCGGTG-3'. dNGLUC was amplified by PCR using primers 5'-GACGAATTCATGCTAGCCAAGCCCACCG-3' and 5'-GGCTACTCTAGGGCACCTGTCCCGCC-3' and sub-cloned into pEAK12-βActin by digestion with *Eco*RI and *Not*I. A DEVDG(2)-Flag sequence was inserted at *Eco*RI as an adapter with the sequences 5'-AATTGGACGAGGTGGACGGCGACGAGGTGGACGGCGACTACAAGGACGA CGACGACAAGGAATTCGC-3' and 5'-GGCCGCGAATTCCTTGTCGTCGTCGTCCTTGTAGTCGCCGTCCACCTCGTC GCCGTCCACCTCGTCC-3' to generate pEAK12-Actin-DEVDG2flag-dNGLUC (DEVDG2F). Similarly, Actin-flagDEVDG2-dNGLUC (FDEVDG2) was constructed by inserting the Flag sequence before the DEVDG2 motif. A mutant Actin-DEVAG2flag-dNGLUC (DEVAG2F) construct was inserted with the same strategy. Actin-DEVDG3-dNGLUC (DEVDG3) was generated by introduction of three adjacent DEVDG sites. The Actin-LC3-dNGLUC construct was generated by PCR of hMAP1LC3 (Origene Trueclone AB2841G10) using primers 5'-GACGAATTCATGCCGTCGGAGAAGAC-3' and 5'-GACGCGGCCGCTTAGGATCCCACTGACAATTTCATCCC-3' and sub-cloned into the *Eco*RI and *Not*I site of pMOWSdSV. dNGLUC was amplified by PCR and inserted into the *Bam*HI and *Not*I site of pMOWSdSV-LC3. The LC3-dNGLUC fusion was transferred by *Eco*RI and *Not*I digestion of pEAK12-β-actin to generate pEAK12-Actin-LC3-dNGLUC. GFP-dNGLUC was constructed by subcloning of dNGLUC into pEAK12-GFP using the *EcoR*I and *Not*I restriction sites. To generate Golgin67-DsRed, Golgin67 (Origene Trueclone AB1045_E08) was amplified by PCR and subcloned into pEAK12-GFP using *Hin*dIII and *Not*I restriction sites. DsRedExpress1 (Clontech, Mountain View, CA, USA) was amplified by PCR and sub-cloned in frame using *EcoR*I and *Not*I restriction sites. Expression vectors for caspase 8 and caspase 9 have been previously described [42]. shRNA vectors for knockdown of human ATG4B (#TRCN0000073801), AKT1 (#TRCN0000010174) and vector control pLKO1 were obtained from Sigma (St. Louis, MO, USA).

### Transfection

293ET cells were cultured in DMEM (supplemented with 10% calf serum plus iron, 0.25 μg/ml gentamycin and 50 μM β-mercaptoethanol) and transfected using calcium phosphate precipitation as described elsewhere [43]. The Origene Trueclone™ cDNA library consisting of approximately 12.000 human expression cDNAs arrayed in 96-well plates were transfected by TransFectin (BioRad, Hercules, CA, USA) along with a GFP expression construct in 293ET cells and screened for morphological changes by fluorescence microscopy (RK and BS, unpublished). Clones displaying signs of cell death were selected for transfection with Actin-DEVDG2F-dNGLUC. Supernatants were harvested after 24-32 h for luciferase analysis. Inhibitors of non-conventional secretion (7 μM Monensin, 10 μg/ml Brefeldin A, 5 μg/ml MG132; all from Sigma) were added 24 h after transfection and medium was collected over a 4 h time period.

### Generation of stable 293ET cell line

Actin-dN and Actin-LC3-dN were subcloned into pMOWS [43] and co-transfected in 293ET cells with expression plasmids for VSV-G and retroviral gag-pol. The medium was changed after 24 h and virus supernatant was harvested and filtered through 0.45 μm filters 48 h after transfection. Untransfected 293ET cells were incubated with retroviral supernatant supplemented with 8 μg/ml polybrene; 48 h later, transduced 293ET cells were selected with puromycin at a concentration of 0.3 μg/ml.

### Western blotting

A polyclonal antibody was raised in rabbit against dNGLUC (Proteintech Group Inc, Chicago, IL, USA). For western blotting, cells were lysed in 1% NP40 lysis buffer (20 mM Tris pH7.4, 150 mM NaCl, 1 mM EDTA, 1 mM ZnCl_2_, 1 mM MgCl_2_, 10% Glycerol) and resolved by SDS-PAGE. Proteins were blotted onto nitrocellulose (BioRad) and immune-stained with antibodies against dNGLUC, GFP (Covance, Princeton, NJ, USA), and Flag M2 (Sigma).

### Confocal microscopy

293ET cells were grown on coverslips and transfected by calcium phosphate precipitations as described. After 24 h, cells were fixed in 4% paraformaldehyde and mounted in aqueous mounting agent (Polysciences, Warrington, PA, USA). Images were obtained using confocal microscopy (BioRad Radiance 2000) and are a flat projection of *z *stacks taken throughout the plane of the transfected cell analyzed by LSM Image software (Carl Zeiss).

### Luciferase and alkaline phosphatase assay

GLUC activity was determined using the Renilla Luciferase kit (Promega, Madison, WI, USA). To avoid harvesting luciferase activity from detached cells, supernatants were spun at 14,000 rpm for 5 minutes. Unless otherwise indicated, 10 μl of supernatant from a 12-well plate (total volume 1 ml) was diluted 1:10 in 100 μl 1 × Renilla lysis buffer and 10 μl of this mixture was added to 100 μl of Renilla substrate prior to analysis in a TopCount luminescence plate reader (Perkin Elmer, Waltham, MA, USA). For 96-well plates, 20 μl of SN was mixed with 20 μl of 2 × Renilla lysis buffer and 50 μl of Renilla substrate was added prior to analysis in the TopCount luminescence plate reader. Cells were lysed in 50 μl of 1 × Renilla lysis buffer and 25 μl of cell lysate was added to 50 μl of Renilla substrate. Secreted alkaline phosphatase was determined using the Phospha-Light™ secreted alkaline phosphatase reporter assay system (Applied Biosystems, Foster City, CA, USA) according to the manufacturer's instructions. Briefly, 50 μl of SN was mixed with 150 μl 1 × dilution buffer and incubated at 70°C for 20 minutes. Diluted SN (50 μl) was mixed with 50 μl of assay buffer and 50 μl of substrate solution before assaying in the TopCount luminescence plate reader.

## Abbreviations

DEVDG2F, Actin-DEVDG2-flagdNGLUC; DEVDG3, Actin-DEVDG3-dNGLUC; ER, endoplasmic reticulum; FDEVDG2, Actin-flagDEVDG2-dNGLUC; FRET, fluorescence resonance energy transfer; GAP, GTPase activating protein; GFP, green fluorescent protein; GLUC, *Gaussia princeps *luciferase; SEAP, secreted alkaline phosphatase; shRNA, small hairpin RNA; SN, supernatant; TBC, Tre/Bub2/Cdc16.

## Authors' contributions

RK designed and performed all the experiments and prepared the manuscript. ZS, KFK and WWH developed the expression library. BS designed and directed the experiments and prepared the manuscript. All authors have read and approved the final manuscript.
